# Whispering gallery mode study of phase transition and shape change in liquid crystal droplets

**DOI:** 10.1039/d5sm01126d

**Published:** 2025-12-30

**Authors:** Arkalekha Neogi, Jaka Zaplotnik, Miha Ravnik, Igor Muševič

**Affiliations:** a Condensed Matter Physics Department, Jožef Stefan Institute Jamova cesta 39 1000 Ljubljana Slovenia igor.musevic@ijs.si; b Faculty of Mathematics and Physics, University of Ljubljana Jadranska 19 1000 Ljubljana Slovenia

## Abstract

We demonstrate that the Whispering Gallery Mode (WGM) lasing spectroscopy is a versatile high resolution tool to study the structure of interfaces of liquid crystalline (LC) droplets immersed in an immiscible fluid, such as water. The eigenfrequencies of WGMs in spherical microcavities are very sensitive to the refractive index profile in the nanometer thin interfacial region. This makes it possible to detect interfacial phenomena and temperature change in LC droplets with interferometric accuracy. We use 10–30 µm diameter droplets of a nematic liquid crystal labeled with a fluorescent dye and floating in water as an optical microcavity that sustains the WGMs. At the isotropic–nematic transition we observe wetting of the droplet's interface by a nanometer-thin layer of paranematic LC. Just below this transition, we observe red-shift and strong fluctuations of WGM spectra just before spherical droplet elongates into a fiber. The experiments are modeled with Finite-Difference Time-domain (FDTD) analysis of WGMs in nematic droplet and we find very good qualitative agreement.

## Abstract 2

Whispering gallery mode spectroscopy was used to detect interfacial phenomena and temperature change in liquid crystal droplets with high resolution. The experiments were modeled with numerical analysis and good qualitative agreement was found.

## Graphical abstract

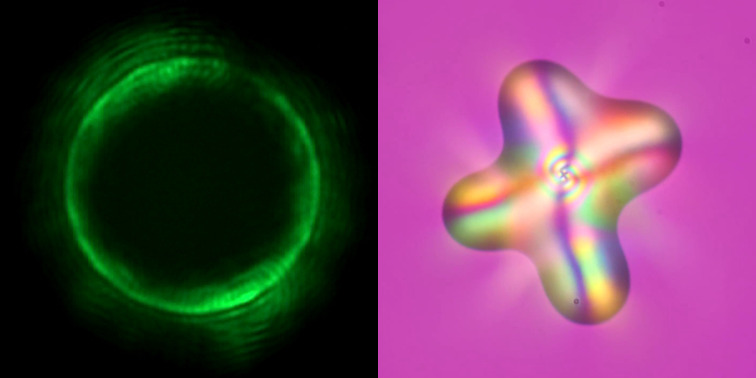

## 1 Introduction

Nematic Liquid Crystals (NLCs) are orientationally ordered fluids,^1^ formed of rod-like molecules that below some temperature (*i.e.* clearing point) spontaneously align along a direction that is called a director. Because of the long range orientational order, the NLCs are uniaxial birefringent fluids that can be elastically distorted and respond to external electric or magnetic fields. The birefringence of NLCs depends on their chemical composition and is in a broad range from ∼0.03 to ∼0.4, whereas the ordinary index of refraction of most NLCs is around ∼1.5.

**Fig. 1:**
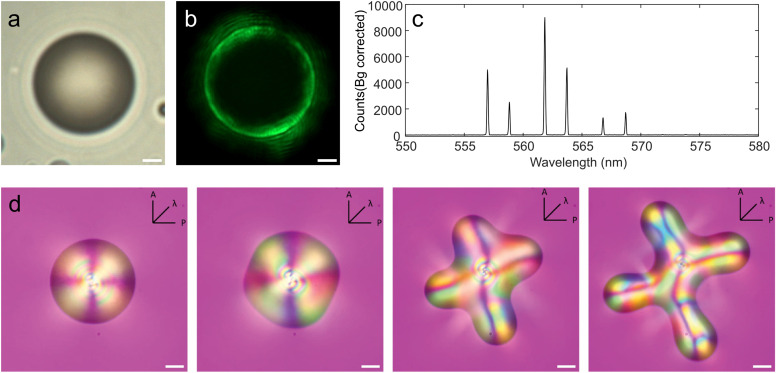
(a) Transmission image of a NLC droplet in isotropic phase with dye. Scale bar 2 µm. (b) Image of a lasing droplet shown in (a). Scale bar 2 µm. (c) Spectra of the lasing droplet in (b). (d) Red plate microscope images of a nematic LC droplet floating in water, which is cooled across the isotropic–nematic phase transition and undergoes shape transformation. The images are taken between crossed polarizers with a lambda (red) plate oriented with the fast axis at 45° with respect to the polarizer (P) and analyzer (A). Scale bar 10 µm.

When the NLC is placed into a confined geometry, such as micrometer-diameter NLC droplets that are floating in some immiscible fluid, the interface of a NLC with the external fluid imposes an orientation of LC molecules, which is either parallel or perpendicular to the surface.^2^Fig. 1(a) shows a NLC droplet that is floating in water with Cetyltrimethylammonium bromide (CTAB) surfactant that provides perpendicular anchoring of NLC molecules at droplet's surface. In terms of optics, such tens-of-micrometer diameter NLC droplets can support optical resonances, known as Whispering Gallery Modes (WGMs). In ray-optics picture, WGMs can be considered as light, circulating inside the micro-droplet by multiple total internal reflections at the interface. These modes are confined very close to the interface, which makes their modal volume very small and very sensitive to the refractive index profile close to the interface. Even a small change of the profile or magnitude of the refractive index close to the interface results in a change of resonance condition, which shifts the WGM spectrum. This sensitivity of WGM resonances to temperature, pressure, force, electric and magnetic fields, gases and biomolecules has been widely exploited in sensor technology.^3,4^ Temperature dependence of the refractive index of the material that forms the WGM microresonator has been widely studied in solid state and soft matter systems, such as silica,^5^ glass,^6^ liquid^7^ and liquid crystals.^8,9^ Based on temperature sensitivity of the WGM material, thermal tuning of WGM cavities has been used for efficient tuning of the coupling the microresonators with waveguides^10^ and for inducing thermal nonlinear effects in very high quality WGM resonators.^11^ WGMs can be made visible by adding fluorescent dye to NLC droplets. This introduces optical gain into the WGM microcavity, which, when optically pumped with an external pulsed laser, eventually results in WGM lasing above some pumping pulse energy, as shown in the photograph in Fig. 1(b). The WGM lasing light can be seen as narrow corona encircling the droplet with typical speckles that indicate spatial coherence of the WGM lasing.

The electric field of a WGM is uniquely characterized by the polarization (TE or TM) and three mode numbers *n*, *l* and *m*, corresponding to the radial, azimuthal and polar directions, respectively.^12,13^ When the NLC is in the isotropic phase, the TE and TM modes experience equal refractive indices, but the modes are slightly split due to the difference in propagation properties of each polarization. This can be seen from the WGM spectrum in Fig. 1(c), which was taken from an isotropic droplet of 4-*n*-Octyl-4-Cyano-Biphenyl (8CB) with a diameter of 10.8 µm at 43°, where one can clearly resolve the TE/TM doublets. When such a droplet is cooled below the isotropic–nematic phase transition (for pure 8CB at 40.5 °C and for our doped 8CB at 36 °C), the NLC droplet adopts the radial director structure, because of the perpendicular molecular orientation at the surface of the droplet. In this phase, the TE and TM modes will experience two different refractive indices.^14^ The electric field of the TM mode is in the radial direction and will experience the extraordinary refractive index *n*_e_ ≈ 1.7, whereas the TE mode will experience the ordinary refractive index of the NLC, which is around *n*_o_ ≈ 1.5. Because of this big difference, the spectrum of the WGM in radial NLC droplets will show beating of their eigenfrequencies.^14^ Molecular orientation in the NLC droplet can be influenced by external electric or magnetic fields, which means that the WGM spectrum from a NLC droplet can be tuned in a range that is nearly two orders of magnitude larger compared to solid WGM microresonators.^14^ It is also well known that the WGM spectrum is very sensitive to molecular absorption at droplet's surface, which alters molecular surface orientation and hence the refractive index at the interface.^15–18^ Because the refractive indices of the NLCs are temperature dependent, WGMs in NLC droplets are also very sensitive to temperature,^19^ which make them interesting for sensing applications. When embedded into an elastic soft material, WGM microresonators made of liquids or liquid crystals can be deformed by forces exerted by the deformable environment. This modifies the WGM spectrum and can be used to measure local stiffness and forces in soft biological tissues.^20^ However, when embedded in liquid environment, such as water, nematic and smectic microdroplets exhibit perfect spherical shape with well defined internal order of LC molecules and high optical quality.

This work was motivated by the recently discovered shape-transformation phenomenon in NLC water dispersions,^21^ which is illustrated in Fig. 1(d). A NLC is doped with a small amount of monoolein surfactant, and dispersed in water with moderate concentration of CTAB surfactant to obtain freely floating droplets shown in Fig. 1(a). Then, the NLC droplet is heated for a few degrees above the clearing point and subject to rapid cooling across the clearing point at a typical rate of ∼1 K min^−1^. When the temperature crosses the clearing point, at some instant the droplet spontaneously starts changing its shape, as can be seen from the second panel in Fig. 1(d). The surface becomes floppy, which is followed either by elongation to a NLC single fiber or ejection of multiple fibers, as illustrated in image sequence in Fig. 1(d). One can conclude from images in Fig. 1(d) that the surface of the NLC droplet has enlarged while crossing the isotropic–nematic phase transition. The spontaneous growth of surface area and the resulting shape transformation must be reflected in the structure of the NLC droplet-water interface and the emergence of orientational order. This opens a question if the onset of the droplet-fibre shape transition could be detected in the corresponding WGM spectra, and what information could be deduced from the WGM spectra, taken during the process of cooling the droplet. The aim is therefore to continuously record WGM lasing spectra from the NLC droplet, while rapidly cooling it across the isotropic–nematic phase transition.

## 2 Experimental setup and procedures

**Fig. 2:**
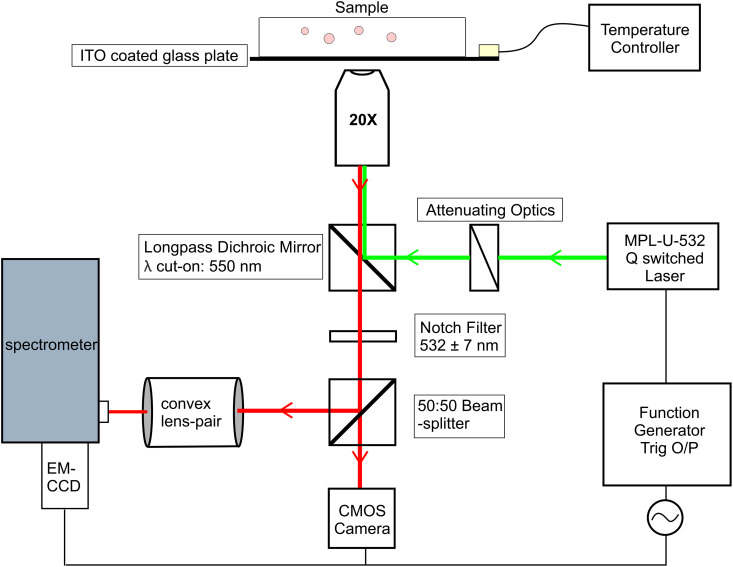
Schematic picture of the experimental setup.

The experimental setup is presented in Fig. 2. The sample was observed using a Nikon TE-2000 inverted polarising microscope, equipped with a 20× objective (Nikon Japan 20X LU Plan), with NA = 0.45. The beam of a Q-switched pulsed laser (CNI optoelectronics, MPL-U-532) operating at the wavelength of 532 nm was passed through a neutral density filter to control the intensity and was directed using a pair of mirrors into the filter cube in the lower turret of the inverting microscope. This filter cube is equipped with a long pass dichroic mirror with cut-on wavelength of 550 nm. The spot size of the pump laser was adjusted by an additional lens to ∼20–40 µm diameter and a typical single pump pulse energy was ∼100 nJ, resulting in a fluence of ∼80–300 pJ µm^−2^ in the focus of the objective. The pumping laser illuminated the edge of the droplet, where the WGMs are located and therefore also excited. The laser pulse energy was measured at the focal plane of the microscope using the Ophir Juno Energy Meter with the Starlab 3.80 software.

The prepared sample is placed on a home built ITO-based temperature stage, controlled by an Oxford Instruments ITC-503 controller for precise temperature control, with the stage temperature monitored using a PT-100 resistor sensor. The light emitted from a selected droplet is collected and passed through the dichroic mirror, which blocks most of the 532 nm laser light. To further eliminate the residual pumping laser signal, the beam then passes through a 532 nm notch filter. The filtered signal is sent to a 50 : 50 beam splitter (400–700 nm) and split between a Flir CMOS camera and an Andor spectrometer. This makes it possible to simultaneously take video images of the droplet and the corresponding WGM spectra. A lens pair between the beam splitter and spectrometer as well as between the beam splitter and the camera ensured that both instruments shared the same focal plane. The spectra of the light, emitted by the selected and lasing NLC droplet, was measured using an imaging spectrometer with a 0.05 nm resolution (Andor, Shamrock SR-500i) and cooled EM-CCD camera (Andor, Newton DU970N). The spectra were collected simultaneously with the laser pulse from the edge volume of the droplet, as illustrated in Fig. 2. A diffraction grating with 1200 lines per mm was used to record spectra in Full Vertical Binning mode (FVB) to maximize the signal-to-noise ratio.

100 µm thick glass cells were made with cover glass of thickness 130–170 µm, separated by 100 µm plastic spacers to ensure the cell gap. We prepared droplets using the 4′-octyl-4-biphenylcarbonitrile (8CB) NLC, which exhibits the isotropic–nematic phase transition at 40.5 °C and nematic-smectic A phase transition at 34 °C. The weights of materials required to prepare a 2 wt% solution of 4′-octyl-4-biphenylcarbonitrile (8CB) doped with 1-oleoyl-rac-glycerol (abbreviated as MO) were calculated and weighed into an amber glass vial. Amber glass was preferred, as MO is light sensitive. Acetone was then added to dissolve the monoolein and the liquid crystal, and the mixture was then heated overnight at 60 °C to evaporate the acetone, resulting in a uniformly mixed solution. A 0.2% solution of PM-580 dye was made in acetone and then added to the doped LC mixture. This was again left to evaporate and uniformly mixed dye into the LC phase, which would act as the gain medium to be excited by the 532 nm laser pulse. The prepared mixture of 8CB, MO and dye exhibited the isotropic–nematic phase transition at 36 °C and nematic-smectic A phase transition at 29 °C. The addition of foreign molecules therefore decreases the phase transitions between LC phases for a couple of degrees.

Further, a 10 mM Cetyltrimethylammonium bromide (CTAB) stock solution was prepared by dissolving the appropriate amount of the solvent and DI water in a volumetric flask. 0.4 mM CTAB was made by diluting this stock solution for the preparation of this sample. The 0.4 mM CTAB solution and the 2% MO-doped 8CB were heated to 45 °C on a hot plate. While heated, 1 L of LC phase is added to 1 mL of 0.4 mM CTAB solution and gently shaken by hand. This produces LC droplets of typical diameters in the range of 10–30 µm, which are interesting because: (i) this size of droplet shows shape-change from droplet to fiber, when rapidly cooled across the isotropic–nematic phase transition, and, (ii) droplets of this size readily sustain WGM resonances due to moderate Q-factor of the microcavity. LC droplets that are smaller than ∼8 µm have a Q-factor that is too small to sustain WGM resonances. Thus, a colloidal solution of LC droplets dispersed in an aqueous medium was prepared. The glass cells were filled with this solution while maintaining temperature and sealed with a two-part epoxy glue (UHU Sofortfest Extra Fast) to prevent evaporation and unwanted fluid flow. This sample was placed on the ITO heater, which maintained the same temperature as the hot-plate. Everything must remain heated during the entire sample preparation step to make sure that sudden temperature quenches do not take place as they can lead to unwanted fiber formation.

The pulsed Q switch laser is triggered using a function generator (Rigol DG812), with a square waveform of amplitude 5 V with frequency 10 Hz, and duty cycle of 0.3. The same signal is used to trigger both the Andor Spectrometer and the Flir Camera and thus all the data collection is synchronised. The temperature of the sample is controlled by the ITO heating stage with transparent heater. A small but constant difference exists between the sensor's reading and the true temperature of the stage. At 37 °C, the droplets are observed to be in the isotropic phase. A temperature sweep is programmed into the controller from 37 °C to 34 °C, in 300 seconds. This along with the 10 Hz laser frequency ensures that we record one WGM corresponding to a change of ∼1 mK in the system. Dark background measurement is taken with the spectrograph to establish the noise level. A droplet is chosen in the sample and the laser pulse is directed onto it. The laser pulse excites the lasing of the whispering gallery modes (WGMs) in the droplet at a single pulse energy >100 nJ, corresponding to a fluence of ≥150 pJ µm^−2^. The droplet is adjusted as per the schematic in Fig. 2. The signal of the function generator is turned off and the camera and the spectrometer are configured to take data using external triggering. Recording is started in both the Flir camera and the spectrometer. The temperature sweep and the function generator output are turned on simultaneously to start data acquisition. During a typical temperature sweep across a cooling interval of 3 °C with ∼1 mK temperature step, approximately 3000 spectra are taken, stored and then analyzed.

## 3 Temperature dependence of WGM spectra across the isotropic–nematic phase transition in LC droplets

**Fig. 3:**
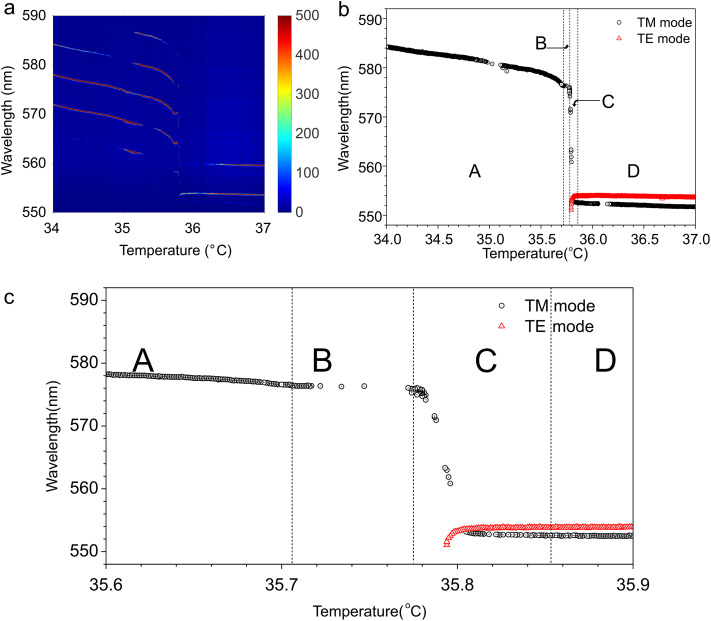
(a) WGM lasing spectrum, emitted from a 9.2 m diameter droplet of 8CB, while cooling at a rate of 0.6 °C per minute. Note the ∼200 mK wide temperature interval between the isotropic and the nematic phase. (b) One of the WGMs extracted from (a). The TM mode is much stronger compared to the TE mode in the nematic phase. (c) Details of temperature dependence of the WGM taken from (b). Note the four characteristic temperature regions.

Fig. 3(a) shows the temperature dependence of WGM spectrum emitted from a 9.2 µm diameter 8CB droplet with monoolein in water with CTAB during cooling at a rate of 0.6 °C per minute. This is a typical cooling rate that induces shape transformation of a NLC droplet into NLC fibres. The shape transformation from a nematic droplet to a nematic fibre is observed for cooling rates from 0.6 K min^−1^ to 30 K min^−1^, and our experiments are at the slower end of the cooling rates to enhance temperature resolution. The whole temperature range of the sweep is 3 K and the phase transition between the isotropic phase of 8CB and the nematic phase of 8CB is at ∼35.0–36.0 °C, depending on the droplet's diameter. Droplets of around ∼25 µm diameter have phase transition at ∼35.0 °C, which increases to 35.8 °C in 8 µm droplets. In the isotropic phase above 36 °C one can clearly see a set of several WGM doublets, each of them corresponds to a pair of TE and TM modes, respectively.^14^ The splitting between the TE and TM modes is of the order of 2–3 nm for the droplet of 9.2 μm diameter and becomes smaller, as the temperature approaches 35.8 °C. At this temperature, the two modes cross each other, the TM mode (“bluish” mode above 36 °C) is red-shifted, whereas the TE mode is blue shifted when the temperature drops below the 36.0 °C. This cross-over is followed by a temperature interval, where the two modes often exhibit non-monotonic, in some cases oscillatory behaviour. Finally, the spectrum clears below 36.7 °C, where one can see a single set of TM modes continuously red-shifting as the temperature is lowered. It is very clear from this data that the behaviour of WGM spectra across the isotropic–nematic phase transition reflects the structural changes near the droplet's interface.

Fig. 3(b) shows a 3 K temperature interval with a single, *e.g.* selected, pair of TE/TM modes. More detailed behaviour of these modes around the phase transition temperature are presented in Fig. 3(c). There are four distinct temperature intervals labeled “A”, “B”, “C” and “D” that can be observed in the experiments. In the “D” region at higher temperature, the entire droplet is in the isotropic phase. At a lower temperature, in the “C” region, the TE/TM pair of modes exhibits mode crossing with red shift of the TM mode and blue shift of the TE mode just close to the transition into the ordered LC phase, clearly seen in Fig. 3(c). This is followed by very strong splitting of the two modes through the region “C”, which is finally stabilized at a plateau “B”. This plateau persists over ∼100 mK, after which there is a gradual red shift of the TM mode in region “A”, whereas the TE mode becomes too weak and disappears from the spectra already in the “C” temperature region.

**Fig. 4:**
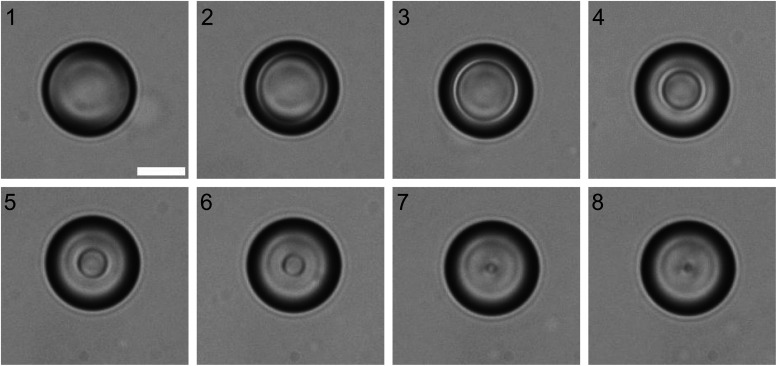
Microscope images of an 8CB droplet with monoolein cooling across the isotropic–nematic phase transition and floating in water with CTAB. Scale bar 10 µm. Numerical labels indicate the temporal sequence, the temperature is dropping from isotropic in 1 to nematic in 8.

This unusual behaviour of the WGMs spectra across the isotropic–nematic phase transition in LC droplets can be understood by considering structural changes in a LC droplet that is cooled across the isotropic–nematic phase transition. Fig. 4 shows a series of images of an 8CB droplet with monoolein that is cooled at a rate of 0.6 °C across the isotropic–nematic phase transition. One can clearly see that while cooling, the nematic order appears as a thin nematic shell around an isotropic core that persists to ∼300 mK below the temperature, where the nematic shell is first observed. The sharp interface between the isotropic and the nematic phase is in agreement with the first order nature of the isotropic–nematic phase transition, where both phases coexist at the temperature of the phase transition. When cooling down in this temperature interval, the thickness of the nematic shell grows until all of the droplet is in the nematic phase. After the droplet completely changes into the nematic phase, the surface becomes floppy and the fibres start growing.

The appearance and thickening of the nematic shell around an isotropic droplet while cooling across the isotropic–nematic phase transition qualitatively explains the observed temperature dependence of the observed WGM spectra. The WGMs are spatially localized at the LC-water interface and therefore the WGM spectrum is very sensitive to the onset of molecular collective orientation and the dielectric tensor in the interfacial layer of the thickness of 300–400 nm. The TM modes have their electric field oscillating in the radial direction and therefore sense the extraordinary index of refraction of the homeotropic LC ordered layer at the interface. The TE modes are sensitive to the ordinary refractive index of this interfacial layer. In the isotropic phase both modes experience the isotropic value of the refractive index of the 8CB liquid crystal. When the temperature of the isotropic–nematic phase transition is reached from above, the thickness of the nematic shell starts growing from zero thickness and grows into the volume occupied by the electric field of the WGMs. This means that the TE mode begins to sense a mixture of isotropic and nematic refractive indices, which grows from purely isotropic value to purely “nematic” value. Here the “nematic” value indicates the extraordinary and ordinary refractive indices at the nematic side of the phase transition. In the first order approximation, one expects that WGMs sense an average value of the refractive index in this mixed region, which means that the TM modes will gradually shift to the red and the TE modes will shift to the blue side of the spectrum, while the thickness of the nematic shell increases from zero to the value that completely occupies the active volume of the WGMs. Once the isotropic–nematic interface completely passes the volume of the WGMs, it will proceed towards the center of the droplet, but the refractive indices will remain constant in the volume occupied by the WGMs.

**Fig. 5:**
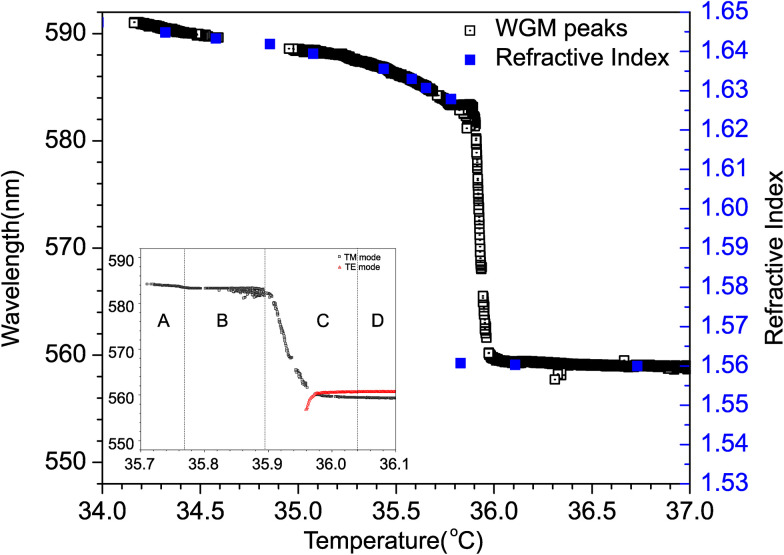
Comparison of the temperature dependence of the extraordinary refractive index of 8CB, taken from ref. 22 (red squares) and the red-shift of one of the WGMs in a 13.4 µm diameter droplet (open circles). The inset shows a zoomed, narrower temperature interval around the phase transition.

The temperature dependence of these refractive indices are well known for 8CB^22^ and is presented in Fig. 5. Note that the temperature scale of *n*(*T*) dependence in this figure is shifted since the phase transition temperature of the used mixture is at ∼36 °C instead of at 40.5 °C as in pure 8CB. In the isotropic phase the refractive index of 8CB is ∼1.565 and slightly increases, as we approach the isotropic–nematic phase transition from above. At the isotropic–nematic phase transition this value splits: the ordinary refractive index discontinuously jumps down to ∼1.53, whereas the extraordinary refractive index jumps up to ∼1.628. The discontinuity in the refractive indices is due to the first-order (*i.e.* discontinuous) nature of the isotropic–nematic phase transition. The discontinuity is accompanied by narrow temperature interval, where both phases coexist, and is around ∼15 mK for pure 8CB in bulk.^23^ In the nematic phase, the extraordinary refractive index continues to increase, whereas the ordinary refractive index continues to decrease. This behaviour of the refractive indices in bulk 8CB explains the general picture of WGM spectra in 8CB droplets while cooling across the isotropic–nematic phase transition at *T*_c_. The TM modes exhibit red shift while crossing and cooling below the *T*_c_, because these modes are sensing the extraordinary refractive index, which increases while cooling down. On the contrary, the TE modes exhibit blue shift while cooling, because they sense the ordinary index of refractive index, which decreases with cooling down. Let us remember that in a simple ray-picture of resonance condition for WGMs implies that 2π*Rn*_eff_ = *l*·*λ*_*l*_. Here *R* is the radius of the droplet, *n*_eff_ is the refractive index that is sensed by the mode, *l* is the mode number and *λ*_*l*_ is the corresponding wavelength of that mode. The wavelengths of the TM modes should therefore shift to the red part of the spectrum with decreasing temperature, because the *n*_eff_ corresponds to the extraordinary refractive index of 8CB that increases with decreasing temperature. On the contrary, the wavelengths of the TE modes should move to the blue because the corresponding refractive index is the ordinary refractive index that decreases with decreasing temperature. This is indeed the general trend, which we observe from the recorded WGM spectra.

Fig. 5 shows a comparison of the temperature dependence of the extraordinary refractive index of 8CB taken from ref. 22 (blue squares) and one of the TM modes. At the phase transition, the jump of the TM mode is comparable to the corresponding jump of bulk extraordinary refractive index. The gradual increase of this index by further cooling is also well reflected in the gradual red-shift of this TM mode.

## 4 Numerical modelling

The behaviour of WGMs at the phase transition in a liquid crystal droplet is numerically analysed using the finite-difference time-domain (FDTD) method^27^ implemented in Meep software.^28^ The studied geometry is a two-dimensional cross-section of an 8CB liquid crystal droplet with radius *R* = 7 µm surrounded with water. The simulation domain is a 19 µm × 19 µm square with open boundary conditions that are provided by 1 μm thick perfectly matched layers (PMLs) which absorb all incoming fields at the domain's boundaries.

**Fig. 6:**
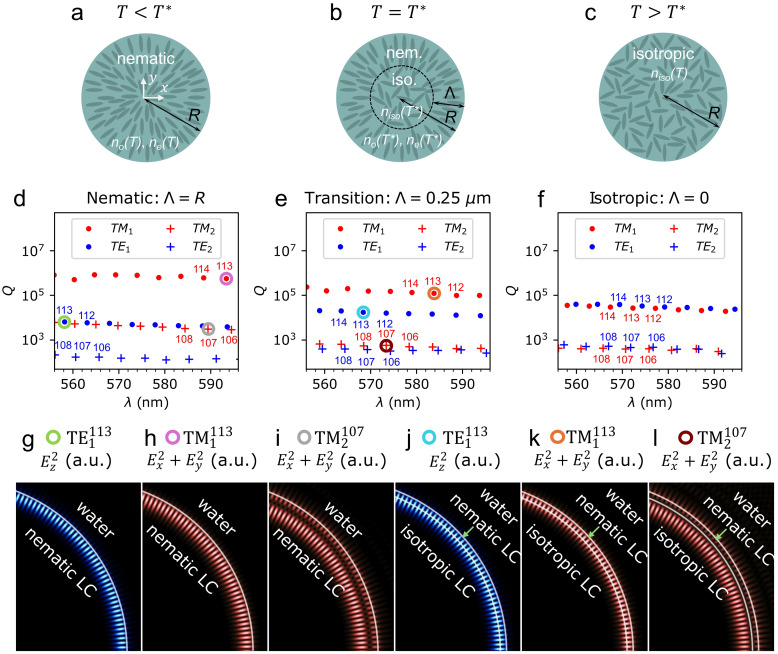
(a)–(c) Structure of the liquid crystal droplet: (a) in the nematic phase, (b) during the phase transition, (c) in the isotropic phase. (d)–(f) Spectra of WGMs *Q*(*λ*) in three different geometries, corresponding to (a)–(c), with *R* = 7 m at the phase transition temperature *T* = T* with refractive indices *n*_o_(T*) = 1.530 and *n*_e_(T*) = 1.628 in the nematic LC domain, *n*_iso_(T*) = 1.562 in the isotropic LC domain and *n*_W_ = 1.33 in water around the droplet. TM modes are depicted in red and TE modes in blue. WGMs with radial mode number *n* = 1 (TM_1_, TE_1_) are marked with circles, while those with *n* = 2 (TM_2_, TE_2_) are marked with crosses. Numerical labels, *e.g.* 112, 113, 114, at selected modes indicate the corresponding azimuthal mode number *l*. (g)–(l) Electric field profiles of selected WGMs in the nematic droplet (g)–(i) and in the droplet with isotropic core and nematic shell of thickness *Λ* = 0.250 µm (j)–(l). *E*_*z*_^2^ is visualised in blue for TE modes and *E*_*x*_^2^ + *E*_*y*_^2^ is visualised in red for TM modes. White curves depict the interfaces between different materials or different phases.

At temperatures below the phase transition temperature T*, the liquid crystal droplet is in the nematic phase and exhibits a radial structure with a +1 radial hedgehog defect in its centre (Fig. 6a). The structure is described by the director field **n**(**r**) = **r**/|**r**|, which does not change with temperature as long as it remains below T*. However, the order parameter varies with temperature, resulting in temperature-dependence of the refractive indices *n*_o_(*T*) and *n*_e_(*T*) (ref. 22). The director field and the refractive indices together define the anisotropic dielectric tensor *ε*_*i*,*j*_(**r**) = *n*_o_^2^*δ*_*ij*_ + (*n*_e_^2^ − *n*_o_^2^)*n*_*i*_(**r**)*n*_*j*_(**r**), where *i*,*j* ∈ {*x*,*y*,*z*}, *δ*_*ij*_ is Kronecker delta, and *n*_*i*_(**r**) are the components of the director field **n**(**r**). At T*, refractive indices in the nematic phase are *n*_o_(T*) = 1.530 and *n*_e_(T*) = 1.628. Conversely, if the temperature is higher than T*, the entire droplet is in the isotropic phase with no orientational order (Fig. 6c), which is characterised by the isotropic refractive index *n*_iso_, or equivalently by the dielectric tensor *ε*_*ij*_ = *n*_iso_^2^*δ*_*ij*_. The isotropic refractive index at the phase transition temperature is *n*_iso_(T*) = 1.562 but it also changes slightly with temperature.^22^

If the droplet is initially in the isotropic phase and is being slowly cooled down – by lowering the temperature of the surrounding water below T*, the nematic order appears at the interface and progresses inwards. In our model, we assume that the ordered nematic domain with a director profile **n**(**r**) = **r**/|**r**| grows uniformly from the interface towards the centre of the droplet. The thickness of the nematic “shell” is denoted by *Λ*, as shown in Fig. 6b. Due to the discontinuous nature of the nematic–isotropic phase transition, the interface between the nematic and the isotropic domain is assumed to be perfectly sharp here. The actual heat-transfer dynamics, and thus the temporal evolution of *Λ*, are not explicitly simulated. Instead, in this model, *Λ* is considered as a geometric parameter, varied between 0.0 µm and 0.9 µm, and we analyze how it affects the wavelengths of the WGMs.

To analyse the spectrum of the liquid crystal droplet's photonic eigenmodes (their eigenfrequencies and corresponding *Q*-factors), we first create a short pulse of light inside the droplet, wait some time for the light with non-resonant frequencies and with resonant frequencies but very low *Q*-factors to propagate out of the droplet, and finally analyse the time-dependence of the remaining resonant electric field ***E***(*t*) in selected points inside the droplet to extract the modes with highest *Q*-factors, that could exhibit low-threshold lasing.

Technically, we generate a pulse of plane waves along a line slightly offset from the symmetry axis of the droplet (centred at *x* = 0, *y* = 0.2 µm), extending over a width of 2.1*R* along the *x*-axis. The generated waves propagate in +*y* and −*y* directions and are polarised along (1,0,1), thereby exciting both TE and TM modes in the droplet. The temporal shape of the pulse is Gaussian with a duration of Δ*t*_FWHM_ ≈ 3.14 fs, corresponding to a spectral width of Δ*λ*_FWHM_ ≈ 34.3 nm. After a delay of 208.3 fs from the pulse maximum – sufficient time for sources to turn off and for non-resonant radiation to escape the droplet – we start recording the time-dependence of the electric field ***E***(*t*) at selected monitoring points for another 66 667 fs. The resonant complex-valued frequencies *ω* (vacuum wavelengths *λ* = 2π*c*_0_/Re(*ω*)) within the spectral width of the pulse and corresponding quality factors *Q* = Re(*ω*)/2 Im(*ω*) are extracted from ***E***(*t*) using the Harminv algorithm, a Fourier-transform-based algorithm implemented in the Meep software.^28^ Specifically, we monitor the *E*_*z*_(*t*) component to analyse TE polarised modes and the *E*_*y*_(*t*) to analyse TM polarised modes, both in two points inside the droplet: (0.05 µm, *R* − *Λ* − 0.05 µm) and (0.05 µm, *R* − 0.05 μm). For each geometry, the simulation is run eight times for eight evenly spaced central wavelengths of the initial pulse within the interval [545 nm, 605 nm] to identify eigenmodes in the whole experimentally relevant range of wavelengths.

To visualise the electric field profile of a selected eigenmode, we tune the central wavelength of the initial pulse to match its eigenfrequency and significantly narrow its spectral width to 0.5 nm, for example, so that only the selected mode is excited. Before visualising the fields, the source amplitude has to decay fully.

## 5 Calculated WGM spectra

Fig. 6a–c illustrates three different geometries in which WGMs are investigated. Corresponding spectra of discrete whispering gallery eigenmodes *Q*(*λ*) are shown in Fig. 6d–f. The spectra are calculated in structures with *R* = 7 µm at a temperature *T* = T* with refractive indices *n*_o_(T*) = 1.530 and *n*_e_(T*) = 1.628 in the nematic LC domain and *n*_iso_(T*) = 1.562 in the isotropic LC domain but in three different stages of the phase transition: in a fully nematic LC droplet (Fig. 6d), in a droplet with an isotropic core and a nematic shell (Fig. 6e), and in a pure isotropic droplet (Fig. 6f).

Based on polarisation, we distinguish between TE modes, in which the only nonzero component of the electric field is *E*_*z*_ (shown in blue), and TM modes, in which *E*_*z*_ = 0 while *E*_*x*_ and *E*_*y*_ are nonzero (shown in red). Additionally, modes are categorized by their radial mode number *n*, which is in *Q*(*λ*) spectra also clearly reflected in the values of *Q*-factors: WGMs with *n* = 1 (TE_1_ and TM_1_) exhibit highest *Q*-factors, followed by modes with *n* = 2 (TE_2_ and TM_2_), *etc.* Another category is the azimuthal mode number *l*, which is related to the wavelength of the mode and is in Fig. 6d–f indicated by numerical labels.

In the isotropic droplet (Fig. 6f), TE and TM modes with the same *n* and *l* appear as doublets in the spectrum:^29^ they exhibit very similar *Q*-factors and wavelengths. The *Q*-factors of TE_1_ are only about 20% higher than those of the TM_1_ modes, while the wavelengths of TE_1_ modes are only about ∼2 nm larger than those of TM_1_ modes with the same *l*.

On the contrary, in the nematic droplet with a radially ordered structure, TE and TM modes with the same *n* and *l* decouple strongly in *Q*(*λ*). The TE polarisation experiences only the ordinary refractive index *n*_o_, while TM polarisation predominantly experiences the extraordinary index *n*_e_.^14^ Consequently, TE and TM modes with the same *l* have significantly different wavelengths: TE modes shift to shorter, and TM modes to longer wavelengths compared to the isotropic case, as shown in Fig. 6d. For example, the wavelengths of the modes TM_1_^113^ and TE_1_^113^ are 572.3 nm and 574.3 nm, respectively, in the isotropic droplet, whereas in the nematic droplet, the wavelength of the TM_1_^113^ is 593.5 nm and 558.4 nm of the TE_1_^113^. Additionally, the refractive index contrast at the LC droplet-water interface also differs for TE and TM modes: it is larger for TM modes (*n*_e_/*n*_W_) than for TE modes (*n*_o_/*n*_W_), which leads to much higher *Q*-factors of TM modes compared to the ones of the TE modes, *e.g.* for TM_1_ modes they are around ∼1 × 10^6^ and for TE_1_ modes only around ∼5 × 10^3^. The spectrum of WGMs *Q*(*λ*) at the phase transition for a nematic shell thickness *Λ* = 0.250 µm is shown in Fig. 6e. It appears as an intermediate stage between the spectra of the fully nematic and fully isotropic droplets. TE and TM modes are already split in wavelengths and *Q*-factors compared to the isotropic case but not as much as in the pure nematic droplet.

When an additional interface is present in the system during the phase transition – between the isotropic core and the nematic shell – one could also expect the emergence of additional WGMs due to internal reflections on this interface. However, for the given geometric and material parameters, the reflectivity at the inner (isotropic–nematic) interface is much lower than at the outer (nematic–water) interface due to larger curvature and smaller refractive index contrast at the inner interface. Consequently, no additional modes with *Q*-factors comparable to the highest *Q*-factors observed in pure nematic or pure isotropic droplets are observed. This clearly suggests that in the investigated geometry, the mode confinement is determined mainly by the outer (nematic–water) interface of the droplet. Furthermore, even electric field profiles of selected modes in the nematic droplet (Fig. 6g–i), and in the droplet with an isotropic core and a nematic shell of thickness *Λ* = 0.250 m (Fig. 6j–l) are qualitatively equal; the growing nematic domain only affects the wavelengths and *Q*-factors of WGMs but does not change the geometry of the mode's electric field profile significantly.

**Fig. 7:**
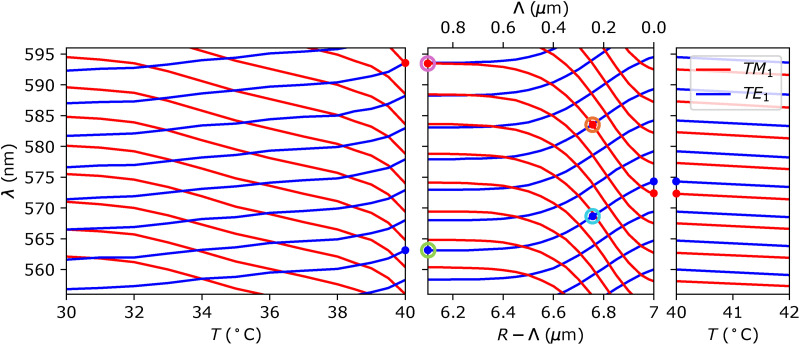
Wavelengths of TM_1_ and TE_1_ WGMs at temperatures around the phase transition temperature T* = 40 °C. Left: TM_1_ and TE_1_ wavelengths in a pure nematic droplet at temperatures below the phase transition temperature T* = 40 °C. Centre: Wavelengths of TM_1_ and TE_1_ WGMs at the phase transition T* = 40 °C at different thicknesses of nematic domain *Λ*. Coloured circles label selected modes which are visualised in Fig. 6. Right: Wavelengths of TM_1_ and TE_1_ WGMs in an isotropic droplet at temperatures above the phase transition temperature T* = 40 °C.

Fig. 7 shows vacuum wavelengths of TM_1_ and TE_1_ WGMs in the 8CB LC droplet at temperatures around the phase transition temperature T* = 40 °C. On the left side of Fig. 7, the spectral shift of the TE_1_ and TM_1_ modes is shown for a pure nematic droplet at different temperatures below T*. The mode wavelengths shift systematically due to the temperature dependence of the refractive indices *n*_o_ and *n*_e_ of the nematic liquid crystal. As temperature decreases, *n*_e_ increases, leading to a redshift of the TM_1_ modes, while *n*_o_ decreases, resulting in a blueshift of the TE_1_ modes. The dependence of refractive indices on temperature is taken from ref. 22.

In contrast, the right side of Fig. 7 corresponds to temperatures above 40 °C, where the whole droplet is in the isotropic phase. In this regime, both TE_1_ and TM_1_ modes experience the same (isotropic) refractive index *n*_I_, which decreases slightly with increasing temperature, leading to a minor blueshift of WGMs of both polarisations.

The central panel of Fig. 7 shows the dependence of WGM wavelengths on the thickness of the nematic domain *Λ* at the phase transition temperature T* = 40 °C. With increasing *Λ* (from right to left), larger and larger part of the WGM profile experiences the nematic (*n*_o_ or *n*_e_) rather than the isotropic refractive index *n*_I_. This causes a blueshift of TE modes and a redshift of TM modes. As *Λ* reaches values ≥0.800 μm, the nematic domain is already thicker than the typical profile of TE_1_ and TM_1_ modes in radial direction (|*E*|^2^(*r*)), and the spectrum of WGMs becomes equal to the spectrum of the pure nematic droplet. Further increase of *Λ*, therefore, does not change the spectrum. The spectrum is most sensitive to changes in nematic domain thickness *Λ* – the derivative |d*λ*/d*Λ*| is largest – at *Λ* ≈ 0.250 μm. Interestingly, at such *Λ*, the boundary between the isotropic and the nematic domain coincides with the centre of the radial profile of TE_1_ and TM_1_ modes, as shown in Fig. 6j and k.

## 6 Discussion

A comparison of the experimental results with numerical calculations shows good qualitative agreement. This makes us confident to propose the following interpretation of WGM lasing experiments on individual NLC droplets while cooling them through the isotropic–nematic phase transition. Far in the isotropic phase, we see WGM doublets that correspond to WGMs with radial mode number *n* = 1 and different polarizations. At a given temperature well above the T*, the TM modes are red-shifted, whereas the TE modes are blue-shifted modes in the doublets. By lowering the temperature, while the droplet is still completely isotropic, the TE/TM modes exhibit a monotonous and slight red shift (∼1 nm °C^−1^, which is due to the temperature dependence of the refractive index in the isotropic phase of 8CB.^22^ However, at about ∼500 mK above the isotropic–nematic phase transition, the splitting between the TE and TM starts decreasing until the modes finally cross each other. The TE mode continues blue-shifting and the TM mode continues red shifting in the nematic phase due to the temperature dependences of the two refractive indices. The TE and TM modes crossing and shifting with decreasing temperature is also clearly seen in numerical calculations in Fig. 7. The crossing point corresponds to the onset of a ∼50 nm thick nematic shell. By lowering the temperature of the heater, the droplets stay at the temperature of the phase transition T*, because the isotropic–nematic phase transition is of first order. This means that at this fixed temperature, the latent heat of the transition is released to the environment (*i.e.* water) and the thickness of the nematic shell is increasing until the whole droplet transforms into the nematic phase. This intermediate region corresponds to temperature intervals labeled “B” and “C” in Fig. 3(c), where the temperature of the droplet is constant in spite of cooling the environment, and the thickness of the nematic shell is growing from the NLC–water interface to the center of the droplet. Finally, we clearly see when the entire droplet has transformed into the nematic phase, as the TE and TM modes start further splitting by reducing the temperature, because the two refractive indices start splitting as well due to the rapidly growing orientational order with cooling.

The mode crossing of the TE and TM modes in the isotropic phase is an interesting observation that cannot be explained by the temperature dependence of the refractive index alone. The mode crossing with decreasing temperature means there are two refractive indices in the “isotropic” phase, which means there is a thin and orientationally at least partially ordered layer of 8CB at the water–LC interface. The LC interfaces show quite often the formation of a thin layer of ordered LC, although far away from the interface the LC is still in the isotropic, *i.e.* disordered phase. This phenomenon is called a pre-wetting (or wetting) of the interface by a thin, orientationally ordered phase, which is in most cases a nematic phase, but in some cases also smectic-like (*i.e.* layered). The order is induced by the ordering potential of the substrate (liquid, solid or gas) and decays exponentially into the isotropic bulk. In many cases, the thickness of this ordered layer increases, and even diverges at the bulk transition temperature, and the interface is completely wetted by the orientationally ordered phase. Wetting by orientational order of a flat interface between 8CB and water with CTAB surfactant has been studied by Ch. Bahr *et al.*^24,25^ using a Brewster angle ellipsometry. The influence of monoolein, which was added to the 9CB LC has also been studied in another ellipsometry study.^26^ Both studies demonstrate the induction of partial orientational wetting of a LC-water interface, when a surfactant is present either in water or in the LC. This nematic pre-wetting is observed in a temperature interval of several degrees in the isotropic phase.^24,25^

**Fig. 8:**
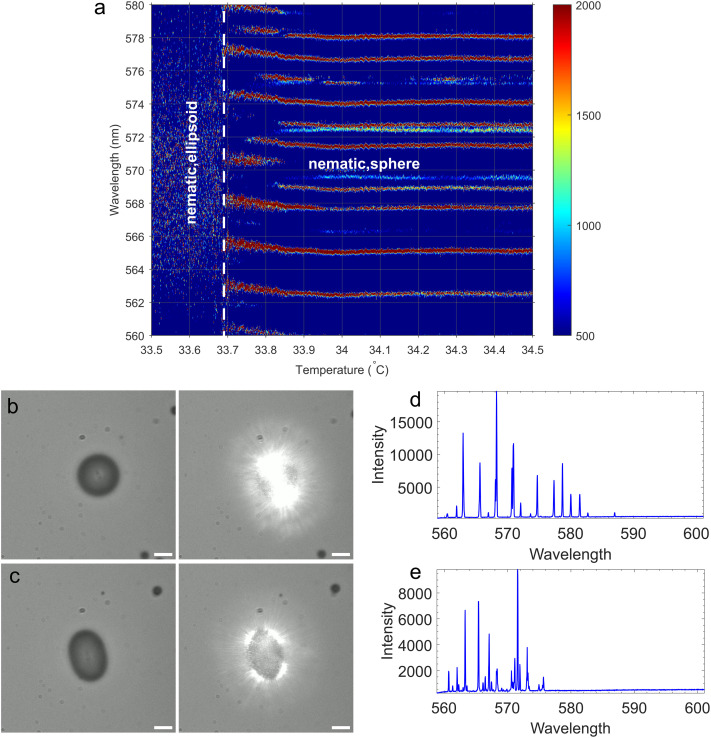
Change of the WGM spectrum across the nematic sphere-ellipsoid shape transition. (a) Temperature dependence of the WGM spectrum in an interval around the shape transformation from a spherical to elliptical droplet. (b) Microscope image of a spherical nematic droplet, together with the image of lasing. (c) Microscope image of an elliptical nematic droplet, together with the image of lasing. (d) WGM spectrum of the spherical nematic droplet shown in (b). (e) WGM spectrum of the elliptical nematic droplet shown in (c).

Finally we show that WGM lasing spectroscopy can detect the onset of the shape change of a nematic droplet from spherical to ellipsoidal. Fig. 8(a) shows temperature dependence of WGM spectra in the spherical nematic droplet (Fig. 8(b), which at ∼33.7 °C (denoted by a vertical dashed line) suddenly starts elongating and transforms into an ellipsoid (Fig. 8(c)), which, by further lowering the temperature, grows into a nematic fibre. One can clearly see a “precursor” of the sphere-fibre shape transformation, as the TM and TE modes gradually shift to the red for ∼1 nm in a temperature interval of ∼200 mK. At the moment fiber starts growing, the spectra changes drastically from a more regular WGM spectrum of a spherical nematic droplet (Fig. 8(d) to the more complex and strongly fluctuating spectrum of the elipsoidal droplet shown in Fig. 8(e). One can also clearly see that just before the shape transformation starts by lowering the temperature, the WGM spectra of spherical droplet becomes noisy, which is a clear indication of fluctuations of the surface area. Namely, at constant mass and volume of the LC, any fluctuation of the surface area will be reflected in the fluctuation of the optical path length, which will in turn result in fluctuations of WGM resonating wavelengths.

## 7 Conclusions

In this work, we demonstrate that WGM spectroscopy is a high resolution and highly sensitive method that can very well resolve surface phenomena in micrometer-diameter droplets of nematic LCs that sustain Mie resonances. By using a fast imaging spectrometer and modest cooling/heating rates, a ∼1 mK temperature step is realistically achievable. On the other hand, mode spectroscopy is an interferometry-based method that is sensitive to small changes of the wavelength of electromagnetic eigenmodes in confined geometry, in our case the precision in measured wavelengths is better than 1 nm. This combination of high temperature and wavelength resolution makes it possible to detect the onset of orientational pre-wetting in isotropic droplets of a nematic LC with precision that is comparable to optical ellipsometry. However, this requires precise measurements of the refractive indices of doped LCs and water with surfactants in a range of temperatures and wavelengths, together with precise measurement of droplet's diameter. Further, we are able to monitor temperature evolution of the orientational order in a droplet that is cooled through the first order isotropic–nematic phase transition. Finally, we demonstrate that WGM lasing spectroscopy is sensitive to any shape change, as this changes the optical path for structural modes. Together with numerical simulation we demonstrated that the WGM spectroscopy is a very powerful tool to study morphological and structural changes in droplets or other structures that sustain structural electromagnetic resonances.

## Conflicts of interest

The authors report there are no competing interests to declare.

## Data Availability

The data that support the findings of this study are available in the Zenodo repository at https://doi.org/10.5281/zenodo.18173648.
